# Effect of fluvoxamine on preventing neuropsychiatric symptoms of post COVID syndrome in mild to moderate patients, a randomized placebo-controlled double-blind clinical trial

**DOI:** 10.1186/s12879-023-08172-5

**Published:** 2023-03-31

**Authors:** Ramin Hamidi Farahani, Ali Ajam, Alireza Ranjbar Naeini

**Affiliations:** AJA University of Medical Sciences, Tehran, Iran

**Keywords:** COVID-19, Fluvoxamine, Post-COVID syndrome

## Abstract

**Background:**

Shortly after the Coronavirus disease 2019 (COVID-19) pandemic, a considerable number of recovered patients reported persisting symptoms, especially neuropsychological manifestations, which were later named post-COVID syndrome (PCS). Immune dysregulation was suggested as one of the main mechanisms for PCS. Fluvoxamine, a selective serotonin reuptake inhibitor (SSRI) that is mostly used to treat depression, anxiety disorders, and obsessive–compulsive disorder, has been suggested as an anti-COVID drug due to its anti-inflammatory effects, mainly through the sigma-1 receptor. Therefore, we aimed to evaluate fluvoxamine's effect on PCS neuropsychiatric symptoms.

**Method:**

In this double-blind randomized clinical trial, we included confirmed mild to moderate COVID-19 outpatients using polymerase chain reaction (PCR) by an infectious disease specialist. The presence of severe COVID-19 symptoms was evaluated by the infectious disease specialist and included dyspnea, SpO2 < 94% on room air, PaO2/FiO2 < 300 mm Hg, a respiratory rate > 30 breaths/min, and lung infiltrates > 50%. Then we performed permuted block randomization and assigned patients 1:1 into two groups to either receive fluvoxamine 100 mg tablet or a placebo daily for 10 days. Eligible patients were evaluated after 12 weeks for the presence of fatigue, as the primary, and other PCS symptoms as secondary outcomes.

**Results:**

We screened a total of 486 patients from March to June 2022. After 12 weeks, 42 patients receiving fluvoxamine and 43 patients receiving Placebo were evaluated for PCS. Patients had a mean age of 38.5 ± 14.1 and 48% of them were women. Fatigue was significantly lower in the fluvoxamine group (*p*-value 0.026). No significant differences were observed in other symptoms.

**Conclusion:**

We concluded that taking fluvoxamine during active COVID-19 can reduce the chance of fatigue but the advantage of fluvoxamine was not observed for other symptoms. Further studies are necessary to confirm these preliminary results.

## Introduction

In the last days of 2019, several cases of pneumonia of unknown etiology were reported to the World Health Organization (WHO) in Wuhan, China, which cause was confirmed to be of the Coronaviridae family a week later and is now called severe acute respiratory syndrome coronavirus 2 (SARS-CoV-2) [1]. The disease spread rapidly around the world and on March 11, 2020, the WHO declared the Coronavirus disease 2019 (COVID-19) outbreak a pandemic [2]. Shortly after the pandemic began, researchers noted the persistence of some of the symptoms including fatigue, headache, and dyspnea even after recovery. Given the history of post-viral symptoms in patients with the middle east respiratory syndrome (MERS) and severe acute respiratory syndrome (SARS) in 2003 and 2012 outbreaks respectively, the presence of such post-viral conditions was suspected for SARS-CoV-2 and long COVID or post-COVID syndrome (PCS) was suggested [3]. PCS is defined as conditions with a wide range of new, returning, or ongoing health problems that patients experience after COVID-19 infection [4–6]. PCS can be present in up to 50–70% of admitted patients even after several months [7]. Neuropsychological manifestations of PCS such as anxiety, sleep disorders, depression, dizziness, headache, and fatigue, have a considerable impact on a patient’s quality of life and can last for months [8].

In a systematic review of PCS symptoms, Nasserie et al. reported that 72.5% [55–80%] of COVID-19 patients had at least 1 persistent symptom after the initial disease. The most common symptoms were fatigue (median frequency of 40%) and sleep disorders (29.4%) [9]. Kamal et al. also reported that the most common neuropsychiatric symptoms of PCS among survivors of COVID-19 were fatigue (72.8%), anxiety (38%), joint pain (31.4%), headache (28.9%), and depression (28.6%) [10].

Several mechanisms have been suggested for PCS. Nalbandian et al. mentioned overlapping categories of severe systemic inflammation, microvascular thrombosis, neurodegeneration, and direct viral infection as possible underlying pathology of neuropsychiatric post-COVID symptoms. They proposed that chronic low-level inflammation in the brain and accumulation of memory T cells can lead to persistent effects of COVID-19 [3]. Mazza et al. also suggested a combination of immune response to SARS-CoV-2 and psychological stressors such as social isolation, the impact of a severe and potentially fatal disease, concerns about infecting others, and stigma as the cause of psychiatric consequences of COVID-19. They noted high levels of Interleukin (IL)-1β, IL-6, Interferon (IFN)-γ, C-X-C motif chemokine ligand 10 (CXCL10), and C–C motif chemokine ligand 2 (CCL2) in COVID-19 patients as signs of T-helper-1 cell activation. Furthermore, unlike SARS and MERS viruses, SARS-CoV-2 causes elevated T-helper-2-secreted cytokines such as IL4 and IL10. These cytokine dysregulations are known causes of neuropsychiatric disorders [11].

Fluvoxamine is a selective serotonin reuptake inhibitor (SSRI) that is mostly used to treat depression, anxiety disorders, and obsessive–compulsive disorder [12, 13]. In addition to its effect on serotonin, it has been recently suggested for COVID-19 treatment [14]. Fluvoxamine affects COVID-19 infection through several potential mechanisms. First, as an SSRI, it inhibits the serotonin transporter in platelets, resulting in reduced platelet aggregation and reducing the chance of microvascular thrombosis. Second, fluvoxamine binds to the sigma-1 receptor, a key mediator in the early steps of virus-induced host reprogramming, thus, interfering with the early steps of virus replication. Sigma-1 receptors also modulate innate and adaptive immune responses and regulate inositol-requiring enzyme 1α (IRE1)-driven inflammation. Hence, fluvoxamine activation of sigma-1 receptors inhibits cytokine dysregulation and inflammation. Fluvoxamine also inhibits melatonin degradation leading to another anti-inflammation effect [15, 16].

As mentioned above fluvoxamine has a probable influence on major pathways contributing to PCS neuropsychiatric symptoms, especially when taken in active disease, thus acting as a prophylactic agent for COVID-19 and PCS. Besides, its main effect as an SSRI can also lead to reduced stressors during infection, contributing to a lower chance of psychiatric problems after recovery [11, 12]. Hence, this trial aims to evaluate the effects of fluvoxamine on the prevention of neuropsychiatric symptoms of PCS.

## Methods

### Design and participants

This randomized clinical trial (RCT) was conducted at Besat hospital, a major referral center east of Tehran, Iran. We enrolled patients above 18 years old with confirmed COVID-19 referring to the infectious disease clinic of the hospital. The confirmation of COVID-19 diagnosis was performed using polymerase chain reaction (PCR) by an infectious disease specialist. Only outpatients with primarily mild to moderate COVID-19 whose onset of symptoms was under 1 week were eligible. The presence of severe COVID-19 symptoms was evaluated by the infectious disease specialist and included dyspnea, SpO2 < 94% on room air, PaO2/FiO2 < 300 mm Hg, a respiratory rate > 30 breaths/min, and lung infiltrates > 50% [17]. We excluded patients with a history of epilepsy, mania, depression, cardiac arrhythmia, hepatic or renal dysfunction, current use of antidepressants, prior hypersensitivity to fluvoxamine, and pregnant women or during lactation. Most of the Iranian population had received at least two doses of the COVID-19 vaccine, therefore we also excluded a minority of patients without vaccination. Participants were provided with written informed consent and baseline information including past medical history and COVID-19 vaccination was gathered. Our study was conducted under Helsinki's declaration and was approved by the ethical committee of AJA university of medical sciences [18].

### Intervention

Patients were randomized using permuted block randomization with blocks of 4, 6, and 8 then assigned 1:1 into two groups (A and B) to either receive fluvoxamine 100 mg tablet or a placebo daily orally for 10 days. We chose this regimen based on previous studies on the effect of fluvoxamine on COVID-19 patients and consultation with a local psychiatrist and pharmacologist about fluvoxamine’s adverse effects on the Iranian population who suggested a 100 mg daily dosage [19]. The principal investigator responsible for the allocation sequence, the physician responsible for patients’ enrolment and final evaluation, the biostatistician responsible for data analysis, and the participants were all blind to assigned groups. Allocation concealment was done using the sequentially numbered containers method. Drugs of either group were prepared in identical containers based on allocation sequence by another collaborator who was not involved in other parts of the study. Patients were contacted after 1 week to assess their compliance. According to studies and guidelines, PCS is usually defined as symptoms present at least 1–3 months after initial infection and sometimes up to 6 months, we tried to pick a median time based on other studies therefore we contacted all eligible patients 12 weeks after enrollment for evaluation of PCS symptoms [6, 7, 9, 20, 21]. Patients were excluded if any adverse effect occurred or the medication was discontinued. During both the 1-week and 12-week follow-ups patients were evaluated for serious symptoms such as dyspnea, confusion, and chest pain.

### Primary outcome

The primary outcome of our study was the presence of fatigue as the most common manifestation of the post-COVID syndrome [9]. It was evaluated 12 weeks after enrollment using Fatigue Assessment Scale (FAS) with scores > 22 as an indication of significant fatigue [22].

### Secondary outcomes

The secondary outcome was the presence of other PCS neuropsychological manifestations which were also evaluated 12 weeks after enrollment. Patients were examined for symptoms including headache, vertigo, confusion, memory impairment, poor concentration, sleep disorder, aggression, anxiety, depression, myalgia, distorted taste and smell, and dizziness [3, 6, 9, 20, 21, 23–26]. PCS was defined as having at least one of the above symptoms. We used the Hamilton Anxiety Scale with scores > 18 as an indication of anxiety, and the Major Depression Index (MDI) with scores > 20 as an indication of depression [27, 28]. We evaluated other neuropsychiatric symptoms subjectively. All these evaluations were performed by a physician during history taking. Finally, results were compared between the two groups to assess the effect of fluvoxamine.

### Statistical analysis

The required sample size was calculated using a formula by Chow et al. for superiority trials. Using type I error rate of 0.05, 40% power, 15% margin, and expected proportion of 50% for fatigue, the primary outcome in both groups was due to having no prior information on the effect of fluvoxamine on fatigue [29]. We performed the per-protocol analysis on the observed data. Categorical variables are presented as frequencies (percentages) and compared using the Chi-square test. Numerical variables were demonstrated as mean ± standard deviation and compared using a T-test. All statistical analyses were conducted by a biostatistician unaware of the patients' assigned group using IBM SPSS version 26. We considered a *p*-value of < 0.05 statistically significant.

## Results

### Demographics

**Fig. 1:**
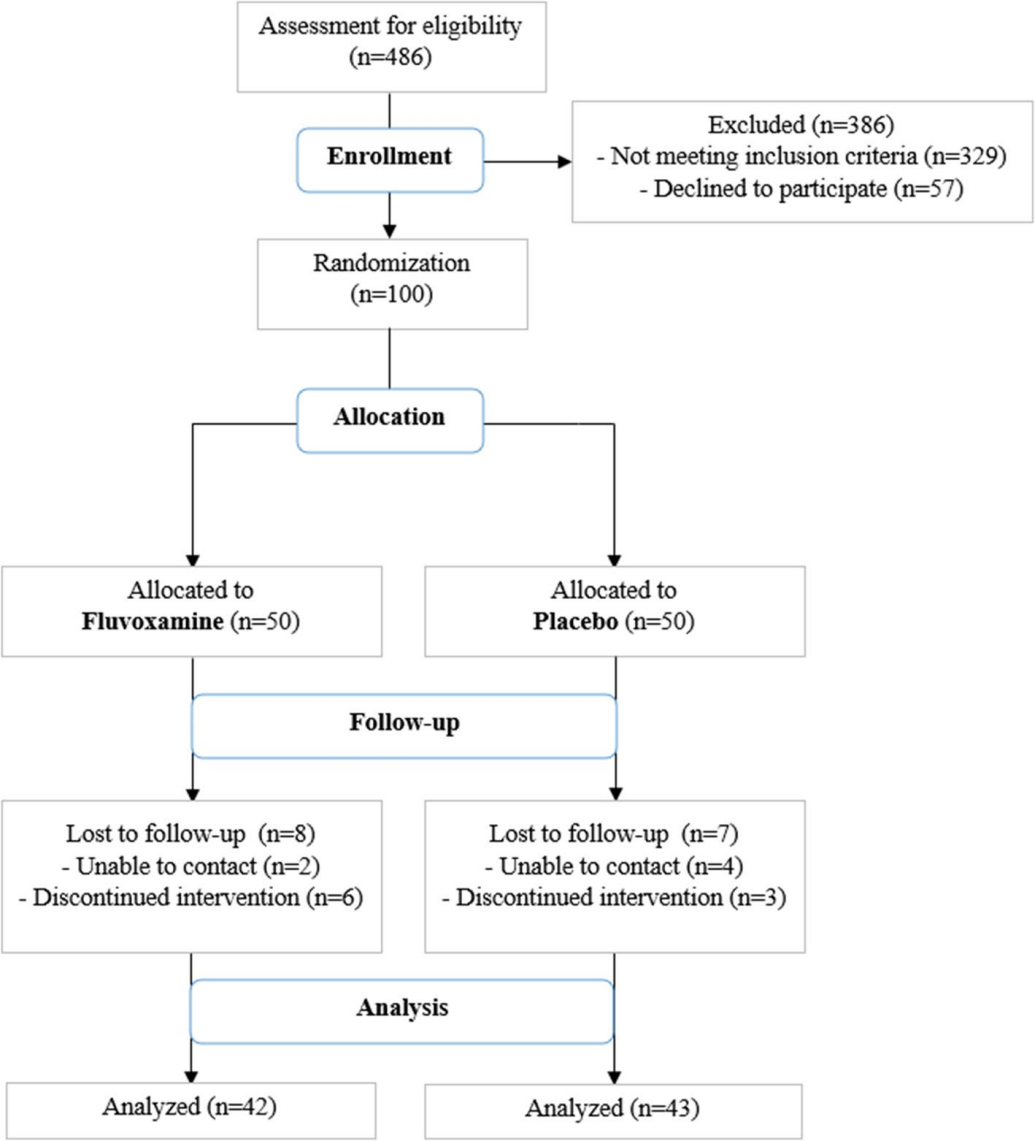
Patients enrollment and follow-up flowchart

We screened a total of 486 patients from March 2022 to Jun 2022, of which 329 patients did not meet inclusion criteria and 57 patients declined to participate in our study. We finally enrolled 100 patients, 50 patients were assigned to fluvoxamine and 50 to Placebo. At 12 weeks of follow-up, 85 patients were eligible for PCS evaluation (42 in fluvoxamine and 43 in Placebo), 9 discontinued medication and we were unable to contact 6 patients (Fig. 1).

Patients had a mean age of 38.5 ± 14.1 (39.8 ± 14 in fluvoxamine and 37.2 ± 14.3 in placebo) and 41 of the patients were women (20 in fluvoxamine and 22 in Placebo). Most of the patients received AstraZeneca (23, 13 in fluvoxamine and 10 in placebo) and Sinopharm (59, 28 in fluvoxamine and 31 in placebo) COVID-19 vaccine. No significant difference was observed between fluvoxamine and placebo groups in terms of baseline characteristics (Table 1).

**Table 1:** Baseline characteristics of patients

Characteristic		Fluvoxamine (*n* = 42)	Placebo (*n* = 43)	*P*-value
Age		39.8 ± 14 [19–70]	37.2 ± 14.3 [18–75]	0.399
Sex				
Women		20 (47.6%)	21 (48.8%)	0.911
Men		22 (52.4%)	22 (51.2%)	
COVID-19 Vaccination				
AstraZeneca		13 (31%)	10 (23.3%)	0.769
Sinopharm		28 (66.7%)	31 (72.1%)	
Sputnik		1 (2.4%)	1 (2.3%)	
Pastocovak		0 (0%)	1 (2.3%)	
Past medical history				
Hypertension		2 (4.8%)	5 (11.6%)	0.713
Ischemic heart disease		3 (7.1%)	2 (4.7%)	0.676
Diabetes mellitus		2 (4.8%)	1 (2.3%)	0.616

### Primary outcome

Neuropsychological symptoms of PCS were present in 76.2% and 81.4% of patients in fluvoxamine and placebo groups respectively. The most common manifestations were fatigue (21.4% in fluvoxamine vs. 44.2% in placebo), anxiety (19% in fluvoxamine vs. 30.% in placebo), and insomnia (21.4% in fluvoxamine vs. 23.3%), and distorted smell (14.3% in fluvoxamine vs. 25.6% in placebo).

**Fig. 2:**
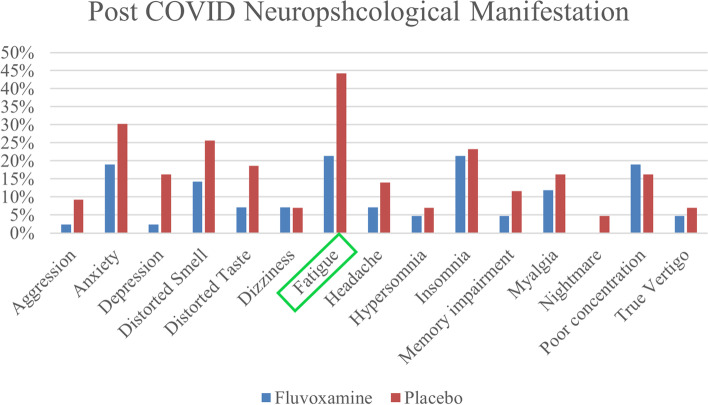
Post-COVID neuropsychological symptoms at 12 weeks after initial infection in fluvoxamine and placebo groups

**Fig. 3:**
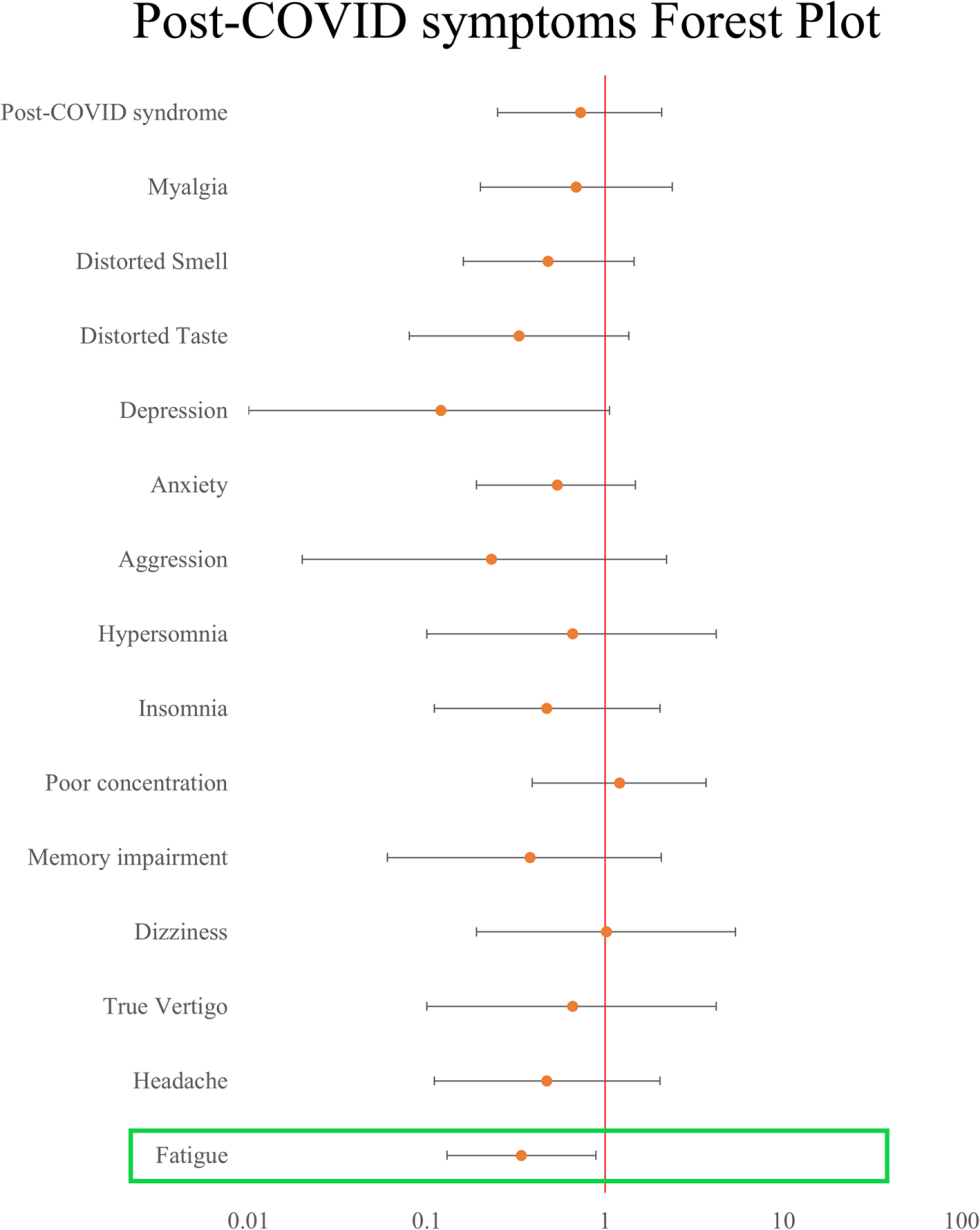
Post-COVID neuropsychological symptoms at 12 weeks after initial infection Forest plot in fluvoxamine and placebo groups using odds ratio

Fatigue was significantly lower in patients receiving fluvoxamine with a relative risk (RR) of 0.48 (95% confidence interval (95% CI):0.25–0.95) and *p*-value = 0.026 (Table 2, Figs. 2 and 3).

**Table 2:** Post-COVID neuropsychological manifestations 12 weeks after the primary COVID-19

Symptom	Fluvoxamine (*n* = 42)	Placebo (*n* = 43)	*P*-value*	Relative Risk
**Primary outcome**				
Fatigue	9 (21.4%)	19 (44.2%)	**0.026**	**0.48 (0.25–0.95)**
**Secondary outcome**				
Headache	3 (7.1%)	6 (14%)	0.483	0.51 (0.14–1.91)
Memory impairment	2 (4.8%)	5 (11.6%)	0.433	0.41 (0.08–1.99)
Poor concentration	8 (19%)	7 (16.3%)	0.738	1.17 (0.46–2.93)
Insomnia	9 (21.4%)	10 (23.3%)	0.840	0.92 (0.41–2.03)
Aggression	1 (2.4%)	4 (9.3%)	0.360	0.25 (0.03–2.19)
Anxiety	8 (19%)	13 (30.2%)	0.232	0.63 (0.29–1.36)
Depression	1 (2.4%)	7 (16.3%)	0.058	0.14 (0.02–1.13)
Distorted Taste	3 (7.1%)	8 (18.6%)	0.115	0.38 (0.11–1.34)
Distorted Smell	6 (14.3%)	11 (25.6%)	0.193	0.56 (0.22–1.37)
Myalgia	5 (11.9%)	7 (16.3%)	0.563	0.73 (0.25–2.12)
PCS	32 (76.2%)	35 (81.4%)	0.557	0.93 (0.75–1.16)

*PCS* Post-COVID syndrome

^*^ Statistically significant *p*-values are bolded

### Secondary outcomes

All other symptoms were less prevalent in patients taking fluvoxamine except for poor concentration (19% in fluvoxamine vs. 16.3% in placebo), Although none were significant. Most considerable one was depression (2.4% in fluvoxamine vs. 16.3% in placebo, *p*-value = 0.058, RR = 0.14 (95% CI: 0.02–1.13)) (Table 2, Figs. 2 and 3).

## Discussion

In this randomized clinical trial, we found out that fluvoxamine reduces the risk of developing fatigue, the most common complaint in PCS when taken during active COVID-19. As for other PCS neuropsychological manifestations, no significant differences were observed between fluvoxamine and placebo, albeit because our primary outcome was fatigue, this trial may not have the power to discuss the effect of fluvoxamine on other post-COVID symptoms.

Although fluvoxamine has been suggested for COVID-19 treatment as early as 2020, to the best of our knowledge this is the first study regarding the effects of fluvoxamine during active infection on post-COVID symptoms [14].

In a study only on COVID-19 outpatients, 53% of COVID-positive patients had persistent symptoms. The Main reported symptoms were fatigue (32%), smell or taste disorder (22%), and headache (12%). They also noted that the prevalence of long-term symptoms was similar during 3–5, 5–7, and 7–10 months after the initial disease [21]. These findings were in line with the findings in our placebo group.

There are several studies on the effect of fluvoxamine but only on COVID-19 severity during initial infection. In 2020 Lenze et al. conducted an RCT on mild COVID-19 patients receiving 300 mg/day of fluvoxamine or placebo for 15 days, evaluating clinical deterioration (shortness of breath or hospitalization for shortness of breath or pneumonia and oxygen saturation less than 92% or need for supplemental oxygen). Clinical deterioration didn’t occur in patients taking fluvoxamine and they had a lower incidence of adverse effects [14]. In the TOGETHER trial on a large population, taking fluvoxamine 200 mg/day for 10 days among high-risk COVID-19 outpatients lead to a reduced need for hospitalization defined as transfer to a tertiary hospital or retention in a COVID-19 emergency setting [30]. In a systematic review by Lee et al. on fluvoxamine treatment for COVID-19 patients, it was concluded that fluvoxamine reduced hospitalization in outpatients with COVID-19 can be recommended as a treatment option, particulate in resource-limited settings [19].

It is noteworthy to mention that the effect of fluvoxamine on depression can be the result of two separate pathways. First the direct effect of fluvoxamine on serotonin levels as an SSRI, and second its probable anti-inflammatory role to prevent post-COVID psychiatric symptoms. We believe that in our study the lower prevalence of depression in patients taking fluvoxamine majorly resulted from the second pathway. The reason for this assumption is that based on the literature, fluvoxamine usually takes 4–6 weeks to effectively treat depression and the duration of the fluvoxamine regimen was 10 days in this trial [31–34]. Nevertheless, this is a preliminary result and further studies are required to further establish this association.

### Limitations

Our study has several potential limitations. First, although conducted in a major referral center, this is a single-center study only on the Iranian population with a relatively small sample size, therefore our findings should be regarded as preliminary. Second, due to worldwide COVID-19 vaccination, future candidates for this drug will probably be vaccinated, patients. In addition, at the time this study was conducted most Iranians received COVID-19 vaccination, therefore there was a very small number of patients who weren’t vaccinated. Thus, we only included patients who had received at least 2 doses of the COVID-19 vaccine to remove this disparity. Consequently, the result of our study may not apply to non-vaccinated patients. Finally, patients in the fluvoxamine group, received a fixed dose of 100 mg/day and higher doses may have a different impact on PCS, thus further studies are required to evaluate the optimal dosage of fluvoxamine. We believe our biggest limitation is the small sample size due to our limited budget, which reduced the power of this study to support the suggested hypothesis and make our study a preliminary and pilot study. We should also mention that because the primary outcome of this study was fatigue, it doesn’t have the power to support finding on other neuropsychological symptoms, our secondary outcomes.

## Conclusion

We concluded that taking fluvoxamine during active COVID-19 can reduce the chance of fatigue as one of the most prevalent post-viral manifestations of SARS-CoV-2. Albeit we couldn’t generalize this result to other neuropsychiatric symptoms of PCS. Finally, due to our limitations, this is a preliminary RCT. We hope this study encourages other researchers to conduct larger trials with alternate doses of fluvoxamine to further evaluate its effect on preventing post-COVID symptoms.

## Data Availability

The dataset generated and analyzed in this study are available from the authors upon reasonable request. Please contact ali.ajam@outlook.com.
